# Data on molecular characterization and expression of the C-reactive protein (CRP) gene from rock bream, *Oplegnathus fasciatus*

**DOI:** 10.1016/j.dib.2019.104506

**Published:** 2019-09-13

**Authors:** Kyung Ho Kim, Do-Hyung Kim, Chan-Il Park

**Affiliations:** a Institute of Marine Industry, College of Marine Science, Gyeongsang National University, 455, Tongyeong, 650-160, Republic of Korea; b Department of Aquatic Life Medicine, College of Fisheries Science, Pukyong National University, Busan, Republic of Korea

**Keywords:** C-reactive protein (CRP), Rock bream, *Edwardsiella piscicida*, *Streptococcus iniae*, Red sea bream iridovirus (RSIV)

## Abstract

C-reactive protein (CRP) is a member of the pentraxin family and is an acute-phase response to plasma protein; its level in blood increases rapidly in response to trauma, inflammation, and infection. In the present study, we analysed the molecular characteristics of the *Oplegnathus fasciatus* CRP (RbCRP) gene sequence using multiple alignments and phylogenetic analyses of the deduced amino acids. In addition, we also examined RbCRP gene expression in rock bream infected with the pathogens *Edwardsiella piscicida* (*E. piscicida*), *Streptococcus iniae* (*S. iniae*) or red sea bream iridovirus (RSIV) and in healthy rock bream individuals. In healthy individuals, RbCRP was ubiquitously expressed in all 14 tested tissues, mainly in the trunk kidney and head kidney. Expression of RbCRP was notably upregulated in the spleen and whole kidney after RSIV infection. This study can provide basic data on the innate immune system of the rock bream to viral and microbial infections.

**Table:** Specifications Table

Subject area	*Immunology and Microbiology*
More specific subject area	*Gene expression analysis*
Type of data	*Figure*
How data was acquired	*GENETYX ver. 7.0. program, Mega version 4.0 and Real-Time PCR*
Data format	*Analysed and Real time PCR*
Experimental factors	*RbCRP gene expression profiles were compared between healthy fish and fish challenged with bacterial and viral infections.*
Experimental features	*Full-length RbCRP cDNA was obtained from expressed sequence tag analysis, and its molecular and expression characteristics were confirmed. This experiment could provide a basis for analysing the functional characteristics of the RbCRP gene in the innate immune system of rock bream.*
Data source location	*Gyeongsang National University, Tongyeong, Republic of Korea*
Data accessibility	*The data are available for this article*

**Table:** 

**Value of the data** • These data provide the possibility of mRNA expression characteristics of RbCRP, which is important in the inflammatory response and induces apoptosis in the rock bream immune system. • These data will provide a basis for understanding the role of RbCRP in the immune system of rock bream infected with various pathogens. • RbCRP mRNA expression analysis Results can be further used for comparative analysis with CRP gene expression assays in teleosts. • These data provide a basis for predicting the function of the CRP gene using tissue-specific expression data of RbCRP and other species of CRP.

## 1 Data

**Fig. 1:**
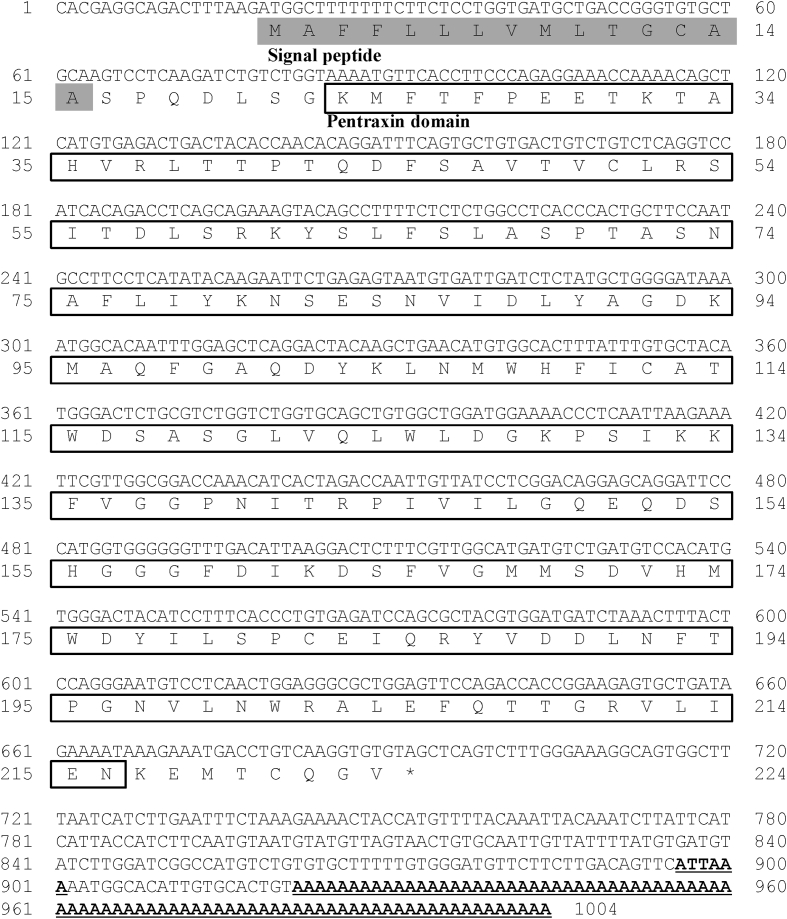
The nucleotide sequence of rock bream CRP (RbCRP) and deduced amino acid sequence. The grey box and box indicate a signal peptide domain and pentraxin domain, respectively. The putative polyadenylation signal (ATTAAA) and a poly-A tail are shown in bold.

**Fig. 2:**
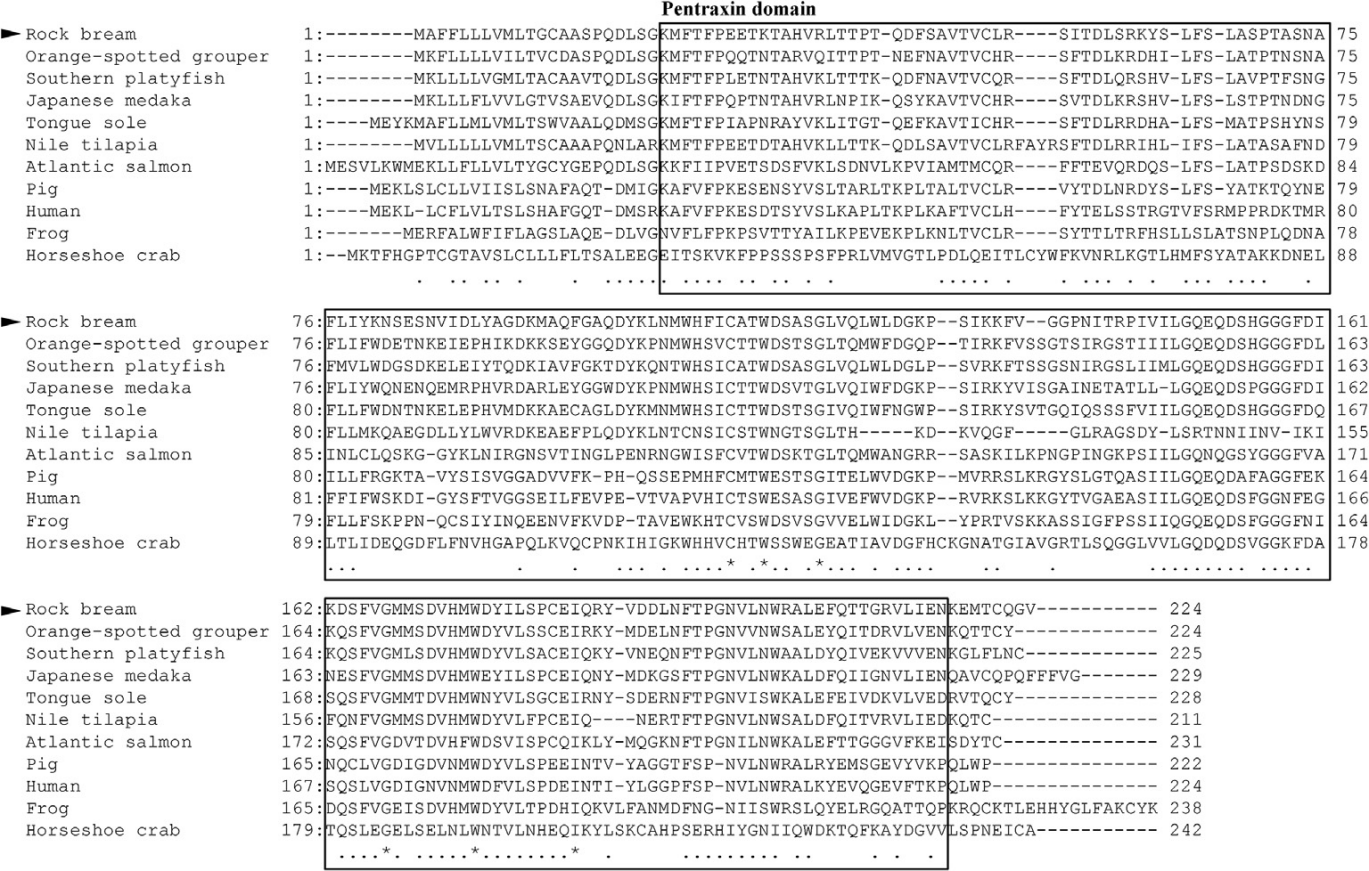
Multiple alignment of amino acid sequences of the rock bream CRP and other species CRP. Amino acids that are identical to the rock bream (*Oplegnathus fasciatus*) sequence are indicated by asterisks (*), similar amino acid residues are indicated by dots (.), and the pentraxin domain is indicated by the box.

**Fig. 3:**
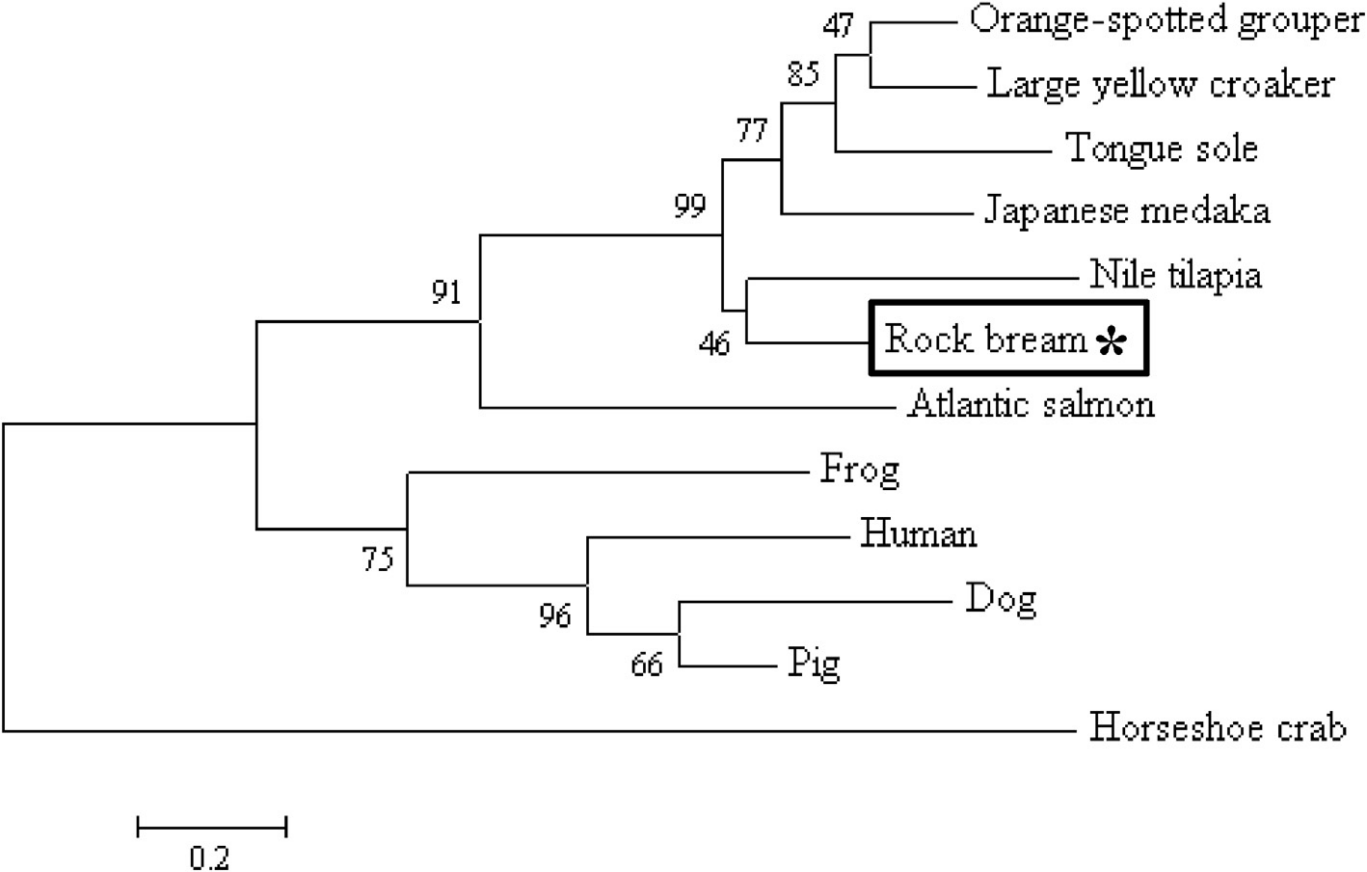
Phylogenetic tree based on amino acid sequences of CRP downloaded from GenBank. The phylogenetic tree was constructed using the neighbour-joining method in MEGA 4 software. Bootstrap sampling was performed with 2,000 replicates. The scale bar represents sequence divergence. GenBank accession numbers: *Salmo salar* (NP_001134140); *Canis lupus familiaris* (CDF47287); *Xenopus laevis* (AAA49692); *Limulus Polyphemus* (AAA28270); *Homo sapiens* (CAA39671); *Oryzias latipes* (XP_004077902); *Oreochromis niloticus* (XP_005472829); *Epinephelus coioides* (ADC92292); *Sus scrofa* (ACF28537); *Cynoglossus semilaevis* (AGD81192); *Larimichthys crocea* (XP_019125679).

C-reactive protein (CRP) has been reported to be an acute-phase response to plasma protein; its level in blood increases rapidly in response to trauma, inflammation, and infection [1], [2]. The open reading frame (ORF) containing the CRP cDNA sequence of rock bream (RbCRP) was identified by expressed sequence tag (EST) analysis of liver tissue from LPS-stimulated rock bream (GenBank accession number: BAM36372). The full-length RbCRP cDNA (1,004 bp) consisted of an 18 bp 5′-untranslated region (UTR), an ORF of 675 bp encoding 224 amino acids, a 311 bp 3′-UTR with a putative polyadenylation signal (ATTAAA), and a poly-A tail. The predicted domains of RbCRP included the signal peptide domain (1 - 15 aa) and the pentraxin domain (23 - 216 aa) (Fig. 1). The isoelectric point and molecular weight of the RbCRP gene were predicted to be 5.32 and 25 kDa, respectively. Multiple alignment analysis of RbCRP amino acid sequences and other species revealed that CRP of Orange-spotted grouper was the most homologous with 65.4% similarity (Fig. 2). A phylogenetic analysis showed that RbCRP was included in the cluster of teleosts and showed the closest affinity to CRP of Nile tilapia (Fig. 3).

**Fig. 4:**
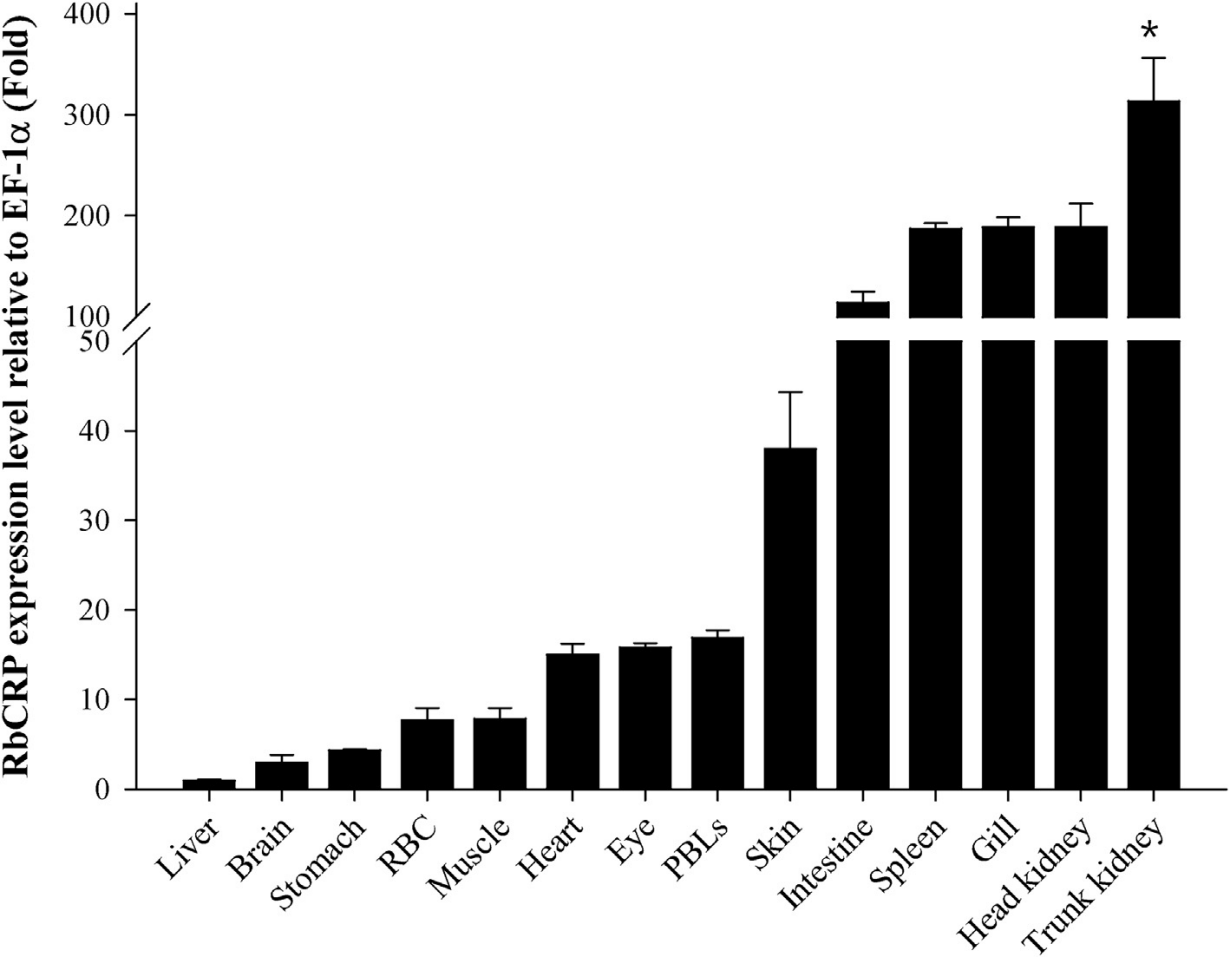
RbCRP mRNA expression in various tissues from healthy rock bream by real-time PCR. EF-1α was used for normalizing the real-time PCR Results. Data are presented as the mean ± SD from three independent cDNA samples with three replicates from each sample. Asterisks indicate significant differences (*P value < 0.05) compared to the liver.

**Fig. 5:**
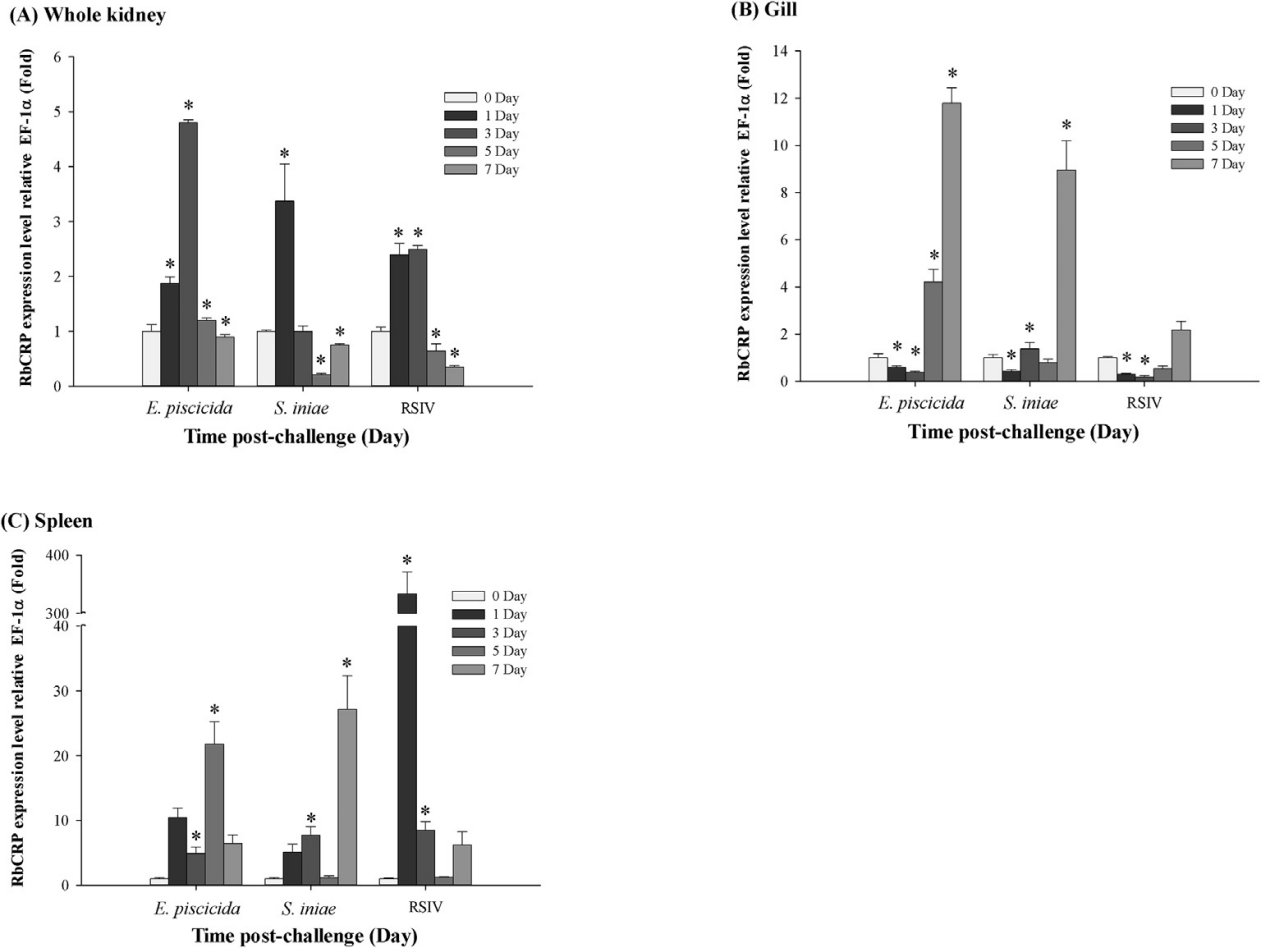
RbCRP mRNA expression levels in (A) whole kidney, (B) the gills, and (C) spleen of rock bream infected with three pathogens [*Edwardsiella piscicida* (*E. piscicida*), *Streptococcus iniae* (*S. iniae*) or red sea bream iridovirus (RSIV)]. The levels of RbCRP transcripts were quantified relative to that of EF-1α levels. The data are presented as the mean ± SD from three independent cDNA samples with three replicates for each sample. The asterisks represent significant differences compared to the control (PBS) group by ANOVA (*P value < 0.05).

Quantitative real-time PCR (RT-qPCR) was used to confirm the expression level of RbCRP mRNA in healthy and pathogen challenged rock bream (*Oplegnathus fasciatus*). As a result of expression analysis of RbCRP mRNA in healthy rock bream, RbCRP mRBA displayed significantly higher expression levels in the trunk kidney (313.7-fold) and head kidney (188.7-fold) compared to the liver (Fig. 4). The expression patterns of RbCRP mRNA in gills, whole kidney, and spleen were confirmed after challenging with *Edwardsiella piscicida* (*E. piscicida*), *Streptococcus iniae* (*S. iniae*) or red sea bream iridovirus (RSIV) in healthy rock bream. After a challenge with RSIV, the expression of RbCRP mRNA was slightly upregulated in the spleen at 1 day and in the spleen and gills at 5 days and 7 days, respectively, after *E. piscicida* infection (Fig. 5).

## 2 Experimental design, materials, and methods

### 2.1 Molecular cloning and sequence analysis

A full-length cDNA of RbCRP was identified from the liver of a lipopolysaccharide (LPS)-stimulated rock bream cDNA library by expressed sequence tag (EST) analysis [3]. The BLAST algorithm of the National Centre for Biotechnology Information (http://www.ncbi.nlm.nih.gov/blast) was used for similarity comparisons with other known amino acid sequences. The domain search of amino acids and of signal peptide prediction were performed using online software on the Simple Modular Architecture Research Tool (SMART) (http://smart.embl-heidelberg.de/) version 4.0 and SignalP web server (http://www.cbs.dtu.dk/services/SignalP/), respectively. The molecular weight and theoretical isoelectric point (pI) values of the proteins was calculated using the ExPASy website (http://web.expasy.org/protparam/).

The GENETYX ver. 7.0 (SDC Software Development, Japan) program (http://clustalw.ddbj.nig.ac.jp/) was used the multiple sequence alignment. The phylogenetic tree was constructed with Molecular Evolutionary Genetics Analysis (MEGA) version 4.0 using the neighbour-joining method with the support of 2000 bootstrap replications [4].

### 2.2 RT-qPCR analysis

#### 2.2.1 Experimental fish and treatment

Healthy rock bream (weighing: 68.5 ± 10 g, body length: 14.3 ± 1 cm) were supplied by the Gyeongsangnam-do Fisheries Resources Research Institute (Tongyeong, Republic of Korea) and maintained at 20–23 °C in aerated seawater until the experiment ended.

To evaluate the level of RbCRP gene expression, healthy rock breams were challenged with an intraperitoneal injection of pathogenic *S. iniae* (3 × 10^6^ cells/fish), *E. piscicida* (2 × 10^6^ cells/fish), or RSIV (1.04 × 10^4^ copies/fish). Control fish were injected with the same volume of phosphate-buffered saline (PBS).

#### 2.2.2 RbCRP gene expression in different tissues of healthy rock bream

To evaluate RbCRP gene expression, various tissues including the liver, brain, stomach, muscle, heart, eye, skin, intestine, spleen gills, head kidney, trunk kidney, peripheral blood leukocytes (PBLs) and red blood cells were isolated from three healthy rock breams. Total RNA was extracted from the various tissues and cells of three healthy rock bream using TRIzol reagent (Invitrogen, USA) according to the manufacturer's instructions. After extraction of total RNA, RNase free DNase (Promega, USA) was used according to the manufacturer's instructions. cDNA synthesis was carried out using the PrimeScript™ 1st strand cDNA Synthesis Kit (Takara, Japan) according to the manufacturer's instructions.

The synthesized cDNA was used as a template for RT-qPCR, which was carried out using a DICE Real-Time System Thermal Cycler (TaKaRa). The RbCRP-specific primer sets were designed by Primer3 ver. 3 based on the cDNA full-length sequence of RbCRP (forward: 5′-TGTGCTACATGGGACTCTGC-3′, reverse: 5′-GCTCCTGTCCGAGGATAAC-3′). The relative mRNA expression levels of RbCRP were calculated using the comparative Ct (2^−ΔΔCT^) method with elongation factor 1 alpha (EF-1 α) (forward: 5′-CCCCTGCAGGACGTCTACAA-3′, reverse: 5′-AACACGACCGACGGGTACA-3′) as a control [5], [6]. The data from each group were tested by an analysis of variance using SPSS 19.0 (IBM, USA). The Results are expressed as the relative fold change compared to the liver.

#### 2.2.3 Expression of RbCRP after challenge with pathogens

The mRNA expression of RbCRP in the whole kidney, spleen, and gills of infected rock bream was measured by RT-qPCR. Each group was sampled from the gills, whole kidney and spleen of the three fish at 1, 3, 5, and 7 days post-injection. All samples obtained were analysed in triplicate, and total RNA extraction, cDNA synthesis and RT-qPCR were performed as described above.

### 2.3 Statistical analysis

Results were assessed using one-way analysis of variance (ANOVA) followed by Fisher's protected least-significant-difference (PLSD) test using SPSS software (ver. 19). For all analyses, a *P* value < 0.05 was taken to indicate statistical significance. All samples were analysed in triplicate; the results are reported as the mean ± standard deviation (SD).
